# Supplementary material for: The Effect of Cold Stress on the Root-Specific Lipidome of Two Wheat Varieties with Contrasting Cold Tolerance
Source: Plants (Basel). 2022 May 20;11(10):1364. doi: 10.3390/plants11101364 (PMC9147729; doi:10.3390/plants11101364)
Supplement: Supplementary file 1 [file plants-11-01364-s001.zip › New Supplemental Table S1_Figures_S1 to S3.pdf]

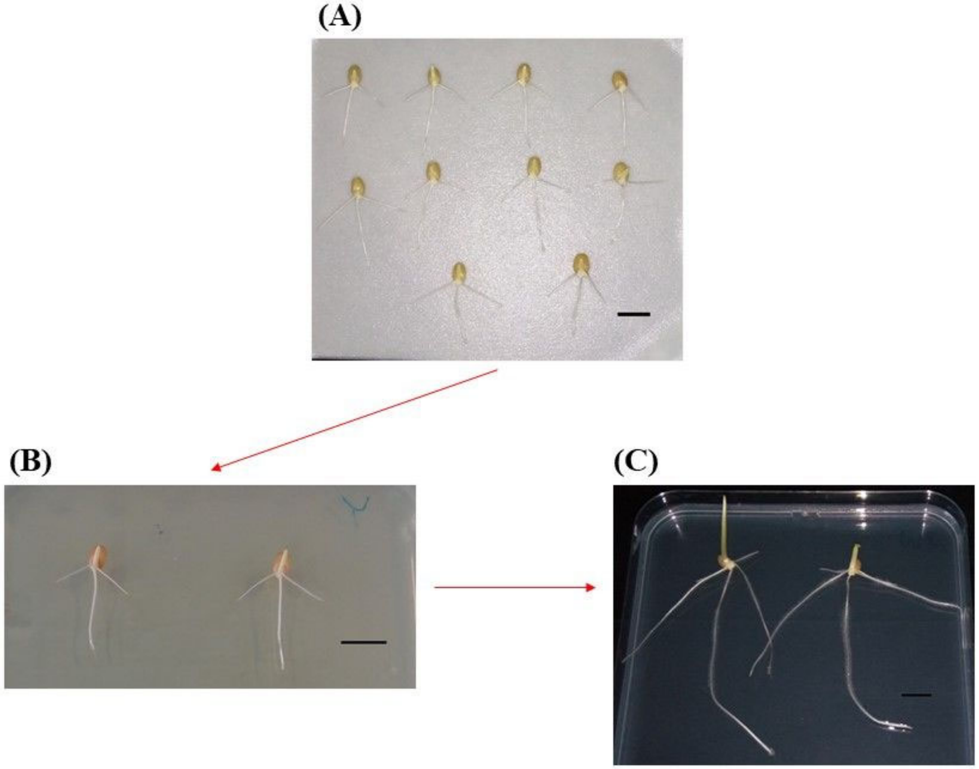

(D)

**20 plates, the germinated seedlings**	**Date and time to put the plates into the 21°C chamber for 48 hrs of growth**	**Date and time to put into the cold chamber (eg. 4°C) for 6 hrs of treatment**	**Date and time to take out the plate from the cold chamber for root sectioning**	The three root zones were immediately snap frozen

**Plate no. 1 and 2**	Day 1, 8 am	Day 3, 8 am	Day 3, 2 pm	with liquid nitrogen after sectioning.
**Plate no. 3 and 4**	Day 1, 8:10 am	Day 3, 8:10 am	Day 3, 2:10 pm	
**Plate no. 5 and 6**	Day 1, 8:20 am	Day 3, 8:20 am	Day 3, 2:20 pm	
**Plate no. 7 and 8**	Day 1, 8:30 am	Day 3, 8:30 am	Day 3, 2:30 pm	
**Plate no. 9 and 10**	Day 1, 8:40 am	Day 3, 8:40 am	Day 3, 2:40 pm	
**Plate no. 11 and 12**	Day 1, 8:50 am	Day 3, 8:50 am	Day 3, 2:50 pm	
**Plate no. 13 and 14**	Day 1, 9 am	Day 3, 9 am	Day 3, 3 pm	
**Plate no. 15 and 16**	Day 1, 9:10 am	Day 3, 9:10 am	Day 3, 3:10 pm	
**Plate no. 17 and 18**	Day 1, 9:20 am	Day 3, 9:20 am	Day 3, 3:20 pm	
**Plate no. 19 and 20**	Day 1, 9:30 am	Day 3, 9:30 am	Day 3, 3:30 pm	

Figure S1. Plant materials and growth conditions for the two wheat varieties used in this study. **(A)** Seeds were germinated on wetted filter paper in dark for 48 hrs at 21°. **(B)** Uniform germinated seedlings with the longest seminal root between 1.5 and 2.0 cm were selected and transferred to square petri dishes containing nutrient agar medium with modified Hoaglands nutrient solution. **(C)** Two seedlings were placed per plate and allowed to grow with the radical orientated downwards by placing the dishes at a 45° angle for another 48 hrs at 21°C in the dark. A biological replicate (n) was the pooled of roots from 20 plates (2 seedlings per plate x 20 plates = 40 seedlings). Only one biological replicate could be harvested or prepared per day. To ensure the cold-treated roots were still cold during the cutting into three root zones, the cold treatment and harvest of roots have to be done in a schedule/cycle as shown in (D). **(D)** A table listing an example of a harvesting schedule/cycle for one biological replicate (20 plates). Bars represent 1 cm.

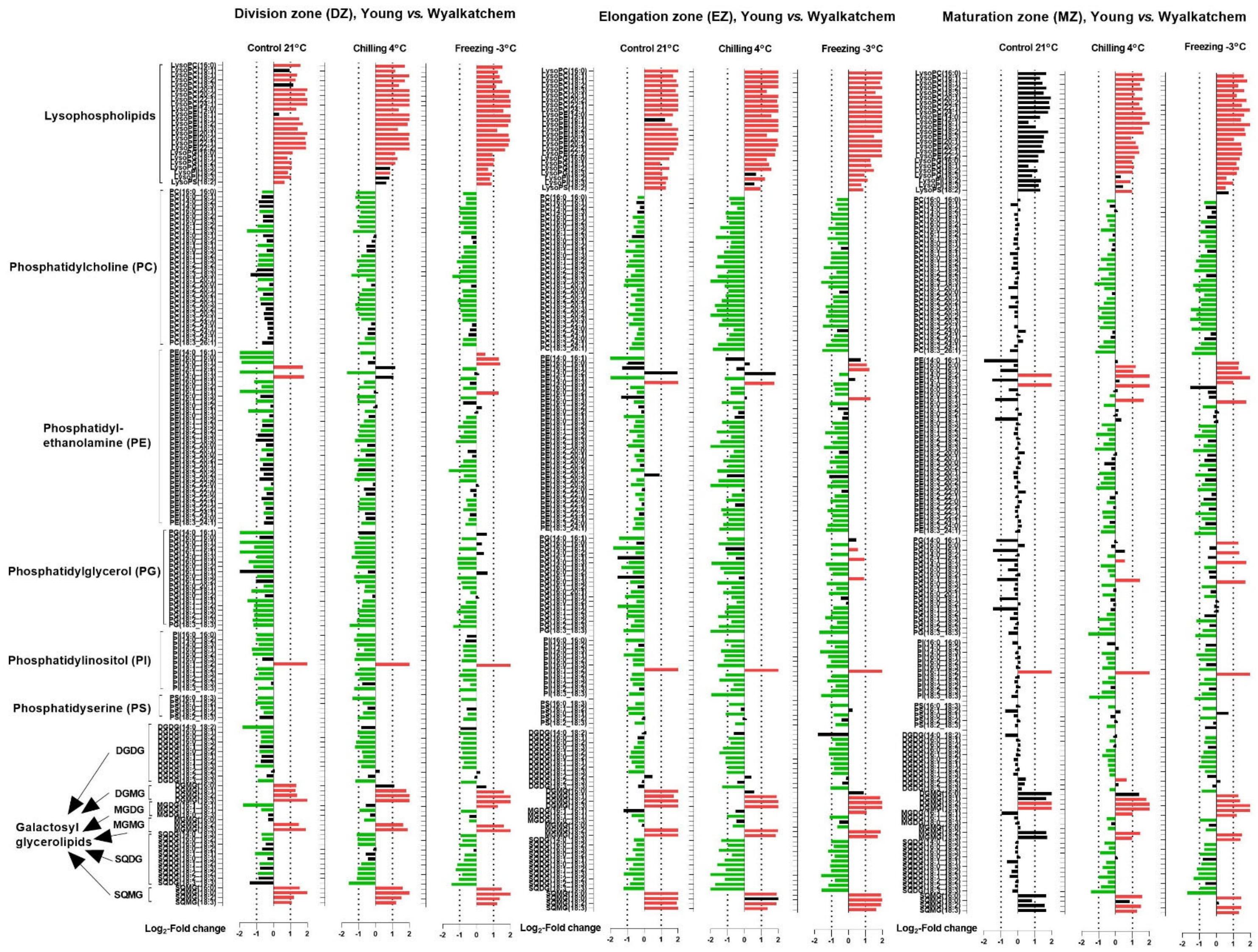

Figure S2. Log2-tranformed of fold changes: Varietal differences of phosphoglycerolipids and galactosyl glycerolipids of the three developmental root zones at the control and after cold stress conditions

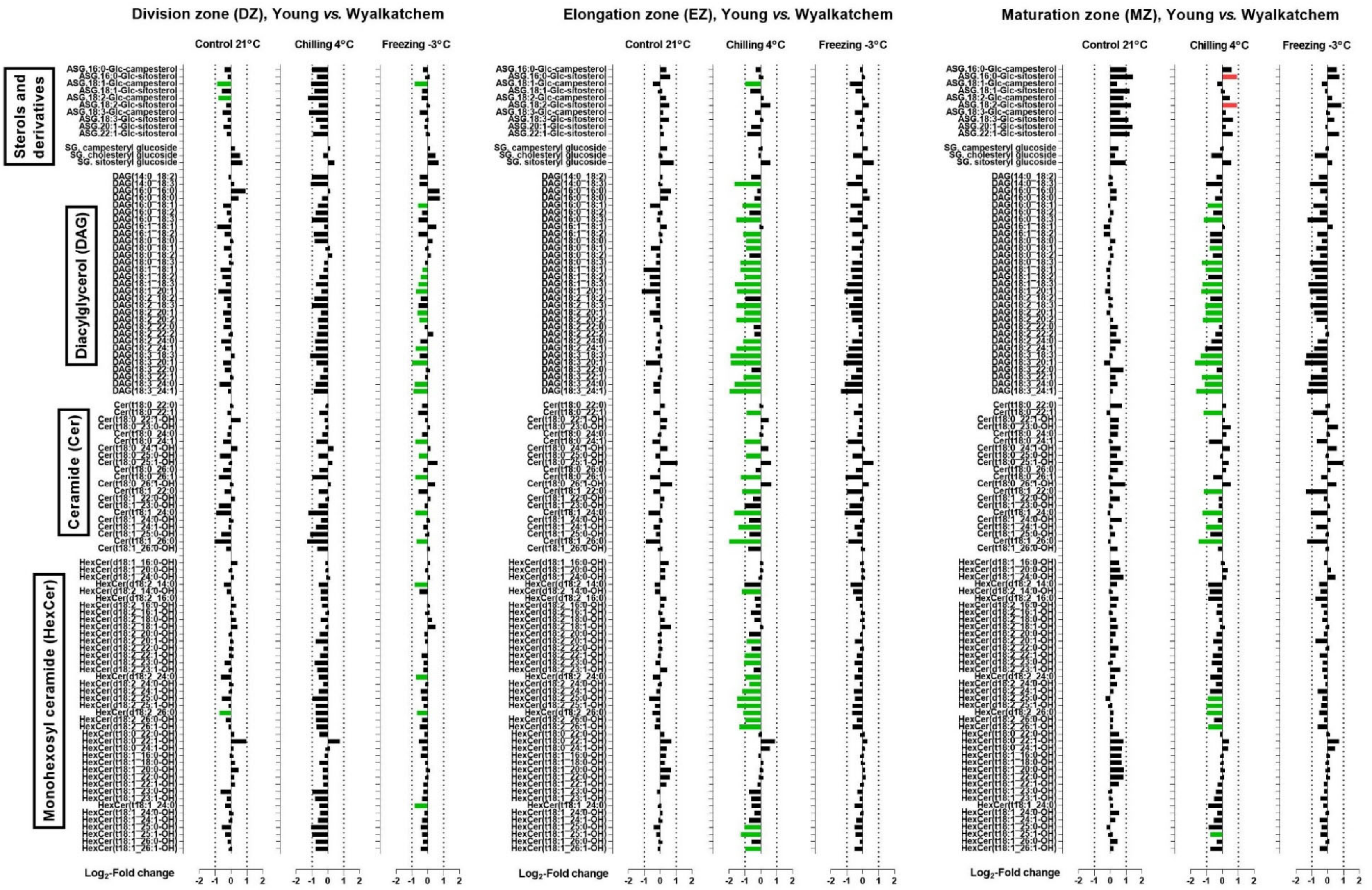

Figure S3. Log2-tranformed of fold changes: Varietal differences of sterols, diacylglycerols (DG) and sphingolipids (Cer and HexCer) of the three developmental root zones at the control and after cold stress conditions.

Table S1. Fold-changes of total 273 lipid species in the three developmental root zones of cold-sensitive Wyalkatchem and cold-tolerant Young at the unstressed stage (control 21°C), after subjected to chilling (4°C) and freezing (-3°C) stress.

	Comparisons in Wyalkatchem						Comparisons in Young						Comparisons between Young vs. Wyalkatchem								
	MZ		EZ		DZ		MZ		EZ		DZ		MZ	EZ	DZ	MZ	EZ	DZ	MZ	EZ	DZ
**Significant x-fold changes (mean) of the 273 lipid species. Significance of differences were determined by FDR-adjusted *p*-value < 0.05 as cut-off and highlighted with yellow colour.**	Chilling 4°C vs Control 21°C	Freezing -3°C vs Control 21°C	Chilling 4°C vs Control 21°C	Freezing -3°C vs Control 21°C	Chilling 4°C vs Control 21°C	Freezing -3°C vs Control 21°C	Chilling 4°C vs Control 21°C	Freezing -3°C vs Control 21°C	Chilling 4°C vs Control 21°C	Freezing -3°C vs Control 21°C	Chilling 4°C vs Control 21°C	Freezing -3°C vs Control 21°C	Control (21°C)	Control (21°C)	Control (21°C)	Chilling (4°C)	Chilling (4°C)	Chilling (4°C)	Freezing (-3°C)	Freezing (-3°C)	Freezing (-3°C)
LysoPC(16:0)	1.0	1.2	1.2	1.7	-1.2	1.1	-1.0	1.2	1.0	1.4	-1.1	1.0	3.2	4.9	3.0	3.0	4.3	3.3	3.1	4.1	2.9
LysoPC(16:1)	-1.4	-1.2	-1.1	1.1	-1.2	1.1	-1.1	1.1	1.1	1.4	-1.0	1.3	2.5	3.3	1.9	3.2	4.2	2.3	3.5	4.3	2.4
LysoPC(18:1)	1.1	1.4	-1.1	1.4	-2.1	-1.6	1.1	1.3	1.0	1.4	-1.4	-1.6	2.7	3.8	2.7	2.7	4.2	4.0	2.4	3.7	2.6
LysoPC(18:2)	1.2	1.4	1.2	1.7	-1.3	1.0	1.1	1.3	1.2	1.7	1.0	1.2	3.2	4.2	2.5	3.0	4.1	3.3	3.1	4.1	2.9
LysoPC(18:3))	1.3	1.5	1.6	2.3	1.0	1.4	-1.0	1.2	1.1	1.9	1.2	1.3	2.9	3.6	2.3	2.2	2.5	2.6	2.3	3.0	2.2
LysoPC(20:1	1.6	1.8	1.2	1.8	-1.5	-1.0	1.2	1.6	1.5	2.3	-1.1	-1.1	3.9	4.5	5.2	3.0	5.7	7.2	3.4	5.6	4.7
LysoPC(20:2)	1.2	1.4	1.4	1.9	-1.1	1.1	-1.1	1.1	1.1	1.9	1.1	1.1	3.6	4.8	3.7	2.6	3.8	4.6	2.8	4.8	3.8
LysoPC(22:1)	1.3	1.3	1.5	2.1	-1.4	-1.3	1.0	1.3	1.4	1.8	-1.1	-1.4	3.7	5.9	4.9	3.0	5.5	6.5	4.0	5.1	4.4
LysoPC(24:1)	1.2	1.4	1.5	1.8	1.0	1.0	1.2	1.3	1.4	1.8	1.1	-1.1	3.4	5.7	5.9	3.4	5.2	6.7	3.1	5.6	5.1
LysoPE(14:0)	-1.3	-1.3	-1.1	-1.2	-1.2	-1.1	-1.0	-1.1	-1.2	1.0	-1.2	1.1	2.5	3.3	2.6	3.0	3.1	2.6	2.7	4.2	3.0
LysoPE(16:1)	-3.3	-3.4	-2.1	-2.8	-2.6	-2.9	1.5	1.9	1.3	1.9	1.3	2.7	1.6	2.4	1.3	7.5	6.4	4.0	10.4	12.4	10.1
LysoPE(18:1)	-1.3	-1.0	-1.3	1.1	-2.1	-1.5	1.1	1.5	1.1	1.6	-1.3	-1.0	2.1	3.1	2.9	3.0	4.5	4.4	3.2	4.4	4.1

LysoPE(18:2)	1.2	1.4	1.2	1.7	-1.2	1.1	1.1	1.3	1.2	1.6	-1.1	1.2	3.4	5.1	3.3	3.1	5.0	3.7	3.3	4.9	3.6
LysoPE(18:3)	1.5	1.8	1.6	2.3	1.0	1.3	-1.0	1.3	1.1	1.7	-1.1	1.1	2.9	3.8	2.7	1.9	2.5	2.5	2.1	2.9	2.4
LysoPE(20:1)	1.2	1.3	1.2	1.7	-1.4	1.0	1.0	1.3	1.2	1.7	-1.2	-1.1	2.8	4.1	4.1	2.3	3.9	4.5	2.8	4.3	3.7
LysoPE(20:2)	-1.1	-1.1	1.1	1.4	-1.4	-1.1	-1.1	1.0	-1.1	1.2	-1.3	-1.1	2.7	4.9	3.7	2.5	4.3	3.9	2.9	4.3	3.9
LysoPE(22:1)	1.1	1.2	1.3	1.5	-1.4	-1.2	1.0	1.2	1.3	1.6	-1.1	-1.2	3.0	3.8	3.8	2.7	3.7	4.8	2.9	3.8	3.7
LysoPE(22:2)	-1.1	-1.2	-1.1	-1.2	-1.4	-1.2	-1.2	-1.1	-1.1	1.0	-1.3	-1.4	2.3	3.4	3.8	2.2	3.5	4.2	2.6	4.1	3.2
LysoPG(16:0)	1.2	1.2	1.3	1.8	-1.1	1.1	1.0	1.2	1.2	1.6	-1.1	-1.0	2.3	2.8	2.2	2.0	2.5	2.2	2.3	2.5	1.9
LysoPG(18:1)	-1.3	-1.1	-1.3	1.1	-1.9	-1.4	1.1	1.5	1.1	1.5	-1.4	-1.2	1.5	1.9	1.8	2.1	2.8	2.5	2.5	2.5	2.1
LysoPG(18:2)	1.2	1.3	1.1	1.5	-1.1	1.1	1.1	1.3	1.3	1.5	-1.1	1.1	2.3	2.8	2.1	2.0	3.0	2.3	2.3	2.8	2.0
LysoPG(18:3)	1.5	1.6	1.6	2.1	1.2	1.4	1.0	1.3	1.2	1.9	-1.0	1.0	1.8	2.1	2.1	1.2	1.6	1.8	1.5	1.9	1.6
LysoPI(18:2)	1.4	1.6	1.3	1.8	-1.1	1.0	-1.0	1.2	1.1	1.4	-1.1	1.1	2.6	2.7	1.8	1.9	2.3	1.8	2.0	2.2	1.9
LysoPI(18:3)	1.6	1.9	1.6	2.2	1.0	1.2	-1.1	1.1	-1.0	1.5	-1.1	-1.0	2.4	2.4	2.1	1.4	1.5	1.8	1.5	1.7	1.7
LysoPS(18:2)	1.4	2.0	1.2	1.8	-1.1	1.0	1.1	1.4	-1.0	1.3	-1.1	1.2	2.5	2.4	1.6	2.0	1.9	1.6	1.7	1.8	1.8
PC(32:0)_PC(16:0_16:0)	1.3	1.8	1.2	1.3	1.2	1.2	-1.2	-1.0	-1.1	1.1	-1.2	1.1	1.1	-1.3	-1.6	-1.4	-1.8	-2.2	-1.7	-1.6	-1.8
PC(32:1)_PC(16:0_16:1)	-1.2	1.1	1.0	1.3	-1.0	-1.0	-1.1	1.0	-1.2	1.0	-1.4	1.0	-1.4	-1.4	-1.6	-1.3	-1.8	-2.3	-1.5	-1.7	-1.5
PC(32:2)_PC(14:0_18:2)	-1.1	1.2	-1.1	1.1	-1.2	-1.0	-1.2	-1.1	-1.2	-1.2	-1.3	1.1	1.1	-1.2	-1.8	1.0	-1.3	-1.9	-1.2	-1.5	-1.5
PC(32:3)_PC(14:0_18:3)	-1.0	1.3	1.3	1.6	1.2	1.3	-1.3	1.0	-1.2	1.1	-1.1	1.1	-1.2	-1.3	-1.8	-1.5	-2.1	-2.2	-1.4	-2.0	-2.0
PC(34:1)_PC(16:0_18:1)	1.2	1.9	1.1	1.3	-1.4	-1.2	-1.0	1.1	-1.2	1.0	-1.5	-1.3	-1.1	-1.6	-1.8	-1.4	-2.0	-1.9	-1.9	-2.1	-2.0
PC(34:2)_PC(16:0_18:2)	1.2	1.7	1.4	1.5	1.1	1.2	-1.0	1.1	-1.1	1.2	-1.1	1.1	-1.0	-1.3	-1.6	-1.3	-1.9	-2.0	-1.6	-1.7	-1.8
PC(34:3)_PC(16:0_18:3)	1.4	1.8	1.6	1.8	1.3	1.4	-1.2	1.1	-1.1	1.2	-1.1	1.1	-1.1	-1.5	-1.6	-1.8	-2.7	-2.1	-1.9	-2.3	-2.0
PC(34:3)_PC(16:1_18:2)	1.0	1.3	1.2	1.3	-1.0	1.1	-1.1	-1.1	-1.1	1.1	-1.0	1.2	-1.1	-1.4	-1.9	-1.1	-1.8	-1.9	-1.5	-1.7	-1.7
PC(34:4)_PC(16:1_18:3)	1.1	1.5	1.7	1.9	1.1	1.1	-1.1	-1.1	-1.1	1.4	1.2	1.7	-1.2	-1.6	-2.9	-1.3	-3.2	-2.5	-2.0	-2.4	-1.9
PC(36:1)_PC(18:0_18:1)	1.1	1.8	-1.2	1.1	-2.5	-1.9	1.2	1.3	-1.1	1.3	-1.8	-1.6	-1.2	-1.9	-1.5	-1.1	-1.7	-1.1	-1.6	-1.5	-1.2
PC(36:2)_PC(18:0_18:2)	1.3	1.8	1.3	1.5	-1.2	-1.0	1.1	1.3	1.0	1.6	-1.1	1.1	-1.0	-1.4	-1.3	-1.2	-1.8	-1.2	-1.4	-1.4	-1.2
PC(36:2)_PC(18:1_18:1)	1.1	1.7	-1.2	1.0	-2.1	-1.7	1.1	1.2	-1.2	1.1	-1.6	-1.5	-1.4	-2.1	-1.9	-1.4	-2.3	-1.5	-1.9	-2.1	-1.7
PC(36:3)_PC(18:0_18:3)	1.4	2.1	1.8	2.1	1.1	1.3	-1.0	1.1	-1.1	1.6	-1.0	1.0	-1.2	-1.6	-1.3	-1.8	-3.0	-1.4	-2.3	-2.0	-1.6
PC(36:3)_PC(18:1_18:2)	1.3	2.0	1.1	1.3	-1.4	-1.2	1.1	1.2	-1.1	1.2	-1.4	-1.2	-1.2	-1.8	-1.9	-1.4	-2.3	-1.8	-2.0	-2.1	-1.8
PC(36:4)_PC(18:1_18:3)	1.4	2.2	1.4	1.7	-1.1	-1.1	-1.1	1.2	-1.2	1.2	-1.3	-1.3	-1.3	-2.0	-1.9	-2.0	-3.3	-2.1	-2.5	-2.7	-2.2
PC(36:4)_PC(18:2_18:2)	1.2	1.7	1.3	1.5	1.0	1.2	-1.0	1.2	-1.0	1.3	-1.0	1.2	-1.0	-1.4	-1.9	-1.3	-2.0	-2.0	-1.5	-1.6	-1.8
PC(36:5)_PC(18:2_18:3)	1.4	1.9	1.7	1.9	1.3	1.4	-1.2	1.1	-1.0	1.5	1.2	1.3	-1.1	-1.7	-2.0	-1.8	-3.0	-2.1	-1.9	-2.2	-2.0
PC(36:6)_PC(18:3_18:3)	1.7	2.3	2.5	2.7	1.8	1.8	-1.2	1.0	-1.1	1.9	1.7	1.7	-1.2	-2.2	-2.6	-2.4	-5.8	-2.7	-2.6	-3.1	-2.7

PC(38:2)_PC(18:1_20:1)	1.3	2.0	1.0	1.2	-1.9	-1.5	1.2	1.2	1.0	1.2	-1.5	-1.6	-1.5	-2.3	-1.9	-1.6	-2.3	-1.5	-2.3	-2.3	-2.0
PC(38:2)_PC(18:2_20:0)	1.1	1.6	1.3	1.4	-1.2	-1.1	-1.0	-1.0	-1.1	1.3	-1.2	-1.2	1.0	-1.3	-1.2	-1.2	-1.9	-1.2	-1.6	-1.4	-1.3
PC(38:3)_PC(18:2_20:1)	1.3	2.0	1.3	1.6	-1.1	1.2	1.2	1.4	1.0	1.4	-1.0	1.2	-1.3	-1.7	-1.9	-1.4	-2.2	-1.8	-2.0	-1.9	-1.8
PC(38:4)_PC(18:2_20:2)	1.4	1.9	1.6	1.7	1.3	1.3	-1.1	1.1	-1.1	1.3	1.0	1.2	-1.1	-1.4	-1.5	-1.6	-2.5	-2.0	-1.8	-1.8	-1.7
PC(38:4)_PC(18:3_20:1)	1.5	2.5	1.8	2.3	1.4	1.6	1.0	1.3	-1.0	1.6	1.2	1.3	-1.4	-1.8	-1.7	-2.0	-3.3	-2.0	-2.9	-2.5	-2.2
PC(38:5)_PC(18:2_20:3)	1.2	1.7	1.6	1.6	1.4	1.5	-1.2	-1.1	-1.4	1.0	-1.0	1.2	-1.1	-1.4	-1.7	-1.7	-3.1	-2.3	-2.3	-2.3	-2.0
PC(38:5)_PC(18:3_20:2)	1.5	2.4	2.2	2.2	2.0	1.8	-1.2	-1.1	-1.1	1.7	1.2	1.3	-1.1	-1.9	-1.4	-2.0	-4.6	-2.3	-2.9	-2.5	-1.9
PC(38:6)_PC(18:3_20:3)	1.4	1.9	1.5	1.7	1.3	1.3	-1.2	1.0	-1.0	1.2	-1.1	1.1	1.0	-1.6	-1.5	-1.6	-2.5	-2.0	-1.9	-2.2	-1.8
PC(40:4)_PC(18:3_22:1)	1.7	2.5	2.2	2.3	1.4	1.5	1.0	1.1	1.4	1.5	1.2	1.3	-1.2	-1.9	-1.5	-1.9	-2.8	-1.8	-2.8	-2.8	-1.8
PC(42:2)_PC(18:2_24:0)	1.4	1.6	1.4	1.5	-1.2	-1.1	-1.1	-1.1	1.1	1.2	-1.1	-1.1	1.4	-1.3	-1.3	-1.1	-1.6	-1.2	-1.3	-1.6	-1.2
PC(42:3)_PC(18:2_24:1)	1.6	2.0	1.9	1.9	1.3	1.2	1.2	1.1	1.4	1.4	1.2	1.2	1.0	-1.4	-1.3	-1.2	-1.8	-1.4	-1.7	-1.8	-1.3
PC(42:3)_PC(18:3_24:0)	1.6	2.2	1.5	1.8	-1.0	1.0	-1.3	-1.0	1.1	1.3	-1.2	-1.2	1.2	-1.5	-1.2	-1.6	-2.1	-1.4	-1.9	-2.0	-1.4
PC(42:4)_PC(18:3_24:1)	1.8	2.4	2.3	2.2	1.6	1.5	1.1	1.2	1.3	1.4	1.3	1.1	-1.2	-1.6	-1.3	-1.9	-2.9	-1.6	-2.4	-2.5	-1.7
PC(44:4)_PC(18:3_26:1)	1.6	2.0	2.0	2.1	1.4	1.2	-1.0	1.0	1.0	1.3	1.1	1.1	-1.4	-1.8	-1.6	-2.3	-3.7	-2.1	-2.7	-2.9	-1.8
PE(30:1)_PE(14:0_16:1)	-3.1	-4.9	-2.0	-3.7	-1.6	-2.0	1.4	2.1	1.2	2.2	2.1	4.6	-4.2	-5.0	-6.4	1.1	-2.2	-1.9	2.5	1.7	1.4
PE(32:0)_PE(16:0_16:0)	-1.7	-1.6	-2.1	-3.0	-2.8	-3.3	1.6	1.8	1.2	1.4	1.4	3.3	-1.2	-1.9	-4.4	2.3	1.2	-1.1	2.5	2.1	2.5
PE(32:1)_PE(16:0_16:1)	-2.8	-3.3	-1.4	-3.5	-1.9	-2.9	1.5	1.8	1.2	1.6	1.4	3.3	-2.1	-2.4	-3.7	2.1	-1.4	-1.4	2.9	2.4	2.6
PE(32:2)_PE(14:0_18:2)	-1.1	1.1	1.1	1.1	1.1	1.1	-1.1	1.6	-1.0	-5.4	-1.4	-5.6	6.6	3.9	3.3	6.1	3.6	2.3	9.2	-1.5	-1.8
PE(32:2)_PE(16:1_16:1)	-2.2	-2.2	-1.5	-1.9	-1.2	-1.7	1.5	2.7	1.2	2.7	2.0	4.6	-2.9	-3.9	-7.9	1.2	-2.2	-3.2	2.0	1.3	1.0
PE(32:3)_PE(14:0_18:3)	1.1	22.4	1.4	1.7	-1.0	1.2	-1.2	1.5	-1.1	-6.0	-1.6	-5.7	5.1	5.3	3.5	4.0	3.4	2.1	-2.9	-1.9	-1.9
PE(34:1)_PE(16:0_18:1)	-1.4	1.1	-1.2	1.0	-1.9	-1.6	-1.0	1.1	-1.2	1.0	-1.4	1.0	-1.4	-1.8	-2.2	-1.0	-1.8	-1.6	-1.4	-1.7	-1.3
PE(34:2)_PE(16:0_18:2)	1.2	1.5	1.3	1.4	1.1	1.2	-1.1	1.1	-1.0	1.1	-1.1	1.1	1.0	-1.3	-1.6	-1.2	-1.8	-2.0	-1.3	-1.7	-1.8
PE(34:2)_PE(16:1_18:1)	-5.8	-5.1	-2.6	-4.6	-3.1	-3.8	1.4	1.7	1.1	1.4	1.3	2.6	-2.5	-2.5	-4.0	3.2	1.1	1.0	3.4	2.5	2.5
PE(34:3)_PE(16:0_18:3)	1.4	2.0	1.6	1.9	1.2	1.3	-1.1	1.1	-1.1	1.3	-1.0	1.1	1.0	-1.5	-1.7	-1.6	-2.5	-2.1	-1.7	-2.2	-1.9
PE(34:3)_PE(16:1_18:2)	-1.2	1.0	1.2	1.3	1.1	1.0	-1.0	1.2	-1.1	1.1	1.1	1.2	-1.1	-1.3	-2.0	1.1	-1.6	-2.1	1.0	-1.5	-1.7
PE(36:2)_PE(18:0_18:2)	1.3	1.7	1.2	1.5	-1.2	-1.2	1.1	1.3	1.0	1.4	-1.1	1.2	1.2	-1.1	-1.1	-1.0	-1.3	-1.0	-1.2	-1.2	1.2
PE(36:2)_PE(18:1_18:1)	-2.6	-1.8	-1.6	-1.4	-2.5	-2.2	1.2	1.4	-1.0	1.2	-1.3	1.2	-2.6	-2.3	-2.8	1.3	-1.5	-1.5	-1.0	-1.3	-1.0
PE(36:3)_PE(18:0_18:3)	1.7	2.7	1.7	2.2	1.1	1.3	-1.0	1.3	-1.0	1.6	1.0	1.0	1.0	-1.4	-1.2	-1.8	-2.5	-1.3	-2.1	-2.0	-1.5
PE(36:3)_PE(18:1_18:2)	1.3	2.2	1.2	1.6	-1.2	1.0	1.1	1.4	-1.0	1.3	-1.3	-1.1	-1.1	-1.5	-1.6	-1.4	-1.8	-1.6	-1.8	-1.8	-1.8
PE(36:4)_PE(18:1_18:3)	1.7	2.6	1.6	2.0	-1.0	1.2	-1.1	1.2	-1.1	1.4	-1.2	-1.2	-1.2	-1.8	-1.6	-2.2	-3.1	-1.9	-2.5	-2.6	-2.1
PE(36:4)_PE(18:2_18:2)	1.4	1.8	1.4	1.5	1.1	1.2	1.0	1.2	1.1	1.3	-1.0	1.2	1.0	-1.3	-1.7	-1.3	-1.7	-2.0	-1.4	-1.6	-1.7

PE(36:5)_PE(18:2_18:3)	1.8	2.3	1.8	2.0	1.2	1.4	1.0	1.3	1.1	1.7	1.2	1.4	1.1	-1.6	-1.9	-1.6	-2.7	-2.0	-1.7	-1.9	-1.9
PE(36:6)_PE(18:3_18:3)	2.0	3.0	2.4	2.7	1.5	1.6	-1.1	1.3	1.1	2.0	1.3	1.5	-1.1	-1.9	-2.1	-2.3	-4.4	-2.3	-2.6	-2.7	-2.3
PE(38:2)_PE(18:2_20:0)	1.3	1.9	1.3	1.5	-1.1	-1.1	-1.1	-1.0	-1.0	1.2	-1.1	1.2	1.3	-1.2	-1.4	-1.0	-1.5	-1.4	-1.4	-1.5	1.0
PE(38:3)_PE(18:2_20:1)	1.3	2.0	1.4	1.6	1.1	1.2	1.2	1.3	1.1	1.3	-1.1	1.3	-1.0	-1.5	-1.6	-1.2	-1.8	-1.8	-1.6	-1.7	-1.4
PE(38:3)_PE(18:3_20:0)	1.5	2.3	1.6	1.9	-1.0	1.0	-1.2	1.0	-1.1	1.2	-1.2	1.1	1.1	-1.4	-1.3	-1.6	-2.4	-1.5	-2.0	-2.1	-1.2
PE(38:4)_PE(18:2_20:2)	1.1	1.4	1.4	1.3	1.2	1.2	-1.1	-1.0	-1.2	-1.0	-1.2	1.2	1.0	-1.2	-1.7	-1.2	-1.9	-2.4	-1.4	-1.6	-1.7
PE(38:4)_PE(18:3_20:1)	1.7	2.7	1.9	2.3	1.3	1.4	1.1	1.4	1.1	1.6	-1.0	1.2	-1.2	-1.7	-1.5	-1.9	-3.0	-2.0	-2.4	-2.5	-1.9
PE(38:5)_PE(18:2_20:3)	1.3	-1.1	2.4	3.8	1.1	1.3	-1.1	-1.4	-1.6	-1.1	-1.0	-1.4	-1.2	1.9	-1.7	-1.6	-2.0	-2.0	-1.6	-2.2	-3.1
PE(38:5)_PE(18:3_20:2)	1.5	1.9	1.8	1.8	1.3	1.2	-1.3	-1.0	-1.2	1.3	-1.1	1.0	1.1	-1.4	-1.5	-1.9	-2.9	-2.1	-1.8	-2.0	-1.7
PE(38:6)_PE(18:3_20:3)	1.5	1.9	2.0	1.9	1.3	1.3	-1.3	1.0	-1.2	1.4	-1.0	1.1	-1.1	-1.7	-1.8	-2.2	-4.2	-2.4	-2.1	-2.3	-2.0
PE(40:2)_PE(18:2_22:0)	1.2	1.6	1.2	1.4	-1.2	-1.1	-1.1	-1.0	1.2	1.2	-1.2	1.1	1.4	-1.1	-1.1	1.1	-1.1	-1.1	-1.2	-1.3	1.1
PE(40:3)_PE(18:2_22:1)	1.5	2.1	1.5	1.6	1.1	1.1	1.3	1.3	1.3	1.3	1.0	1.2	-1.1	-1.4	-1.5	-1.3	-1.7	-1.6	-1.7	-1.8	-1.3
PE(40:3)_PE(18:3_22:0)	1.5	2.3	1.7	2.0	1.0	1.0	-1.1	1.0	-1.0	1.2	-1.1	1.2	1.2	-1.2	-1.3	-1.4	-2.1	-1.5	-1.9	-2.1	-1.1
PE(40:4)_PE(18:2_22:2)	1.0	1.3	1.3	1.4	1.1	1.0	-1.0	-1.0	-1.0	-1.1	-1.1	1.1	-1.0	-1.1	-1.6	-1.1	-1.5	-1.9	-1.4	-1.7	-1.5
PE(40:4)_PE(18:3_22:1)	1.6	2.5	1.8	2.2	1.2	1.1	1.0	1.2	1.0	1.3	-1.1	1.1	-1.1	-1.4	-1.5	-1.7	-2.6	-2.0	-2.3	-2.3	-1.5
PE(40:5)_PE(18:3_22:2)	1.4	1.7	1.6	1.6	1.3	1.2	-1.2	-1.0	-1.2	1.0	-1.1	1.0	-1.1	-1.2	-1.4	-1.7	-2.4	-2.0	-1.8	-1.9	-1.7
PE(42:3)_PE(18:2_24:1)	1.7	2.1	1.7	1.8	1.2	1.1	1.2	1.2	1.4	1.4	1.1	1.3	1.1	-1.5	-1.5	-1.3	-1.9	-1.5	-1.6	-1.9	-1.3
PE(42:3)_PE(18:3_24:0)	1.7	2.3	1.7	2.0	-1.1	-1.0	-1.2	-1.0	1.0	1.2	-1.1	-1.0	1.2	-1.4	-1.4	-1.6	-2.4	-1.5	-2.0	-2.3	-1.4
PE(42:4)_PE(18:3_24:1)	2.1	3.1	2.3	2.4	1.5	1.4	1.2	1.3	1.3	1.6	1.3	1.3	-1.0	-1.6	-1.5	-1.8	-2.8	-1.8	-2.5	-2.5	-1.6
PG(30:1)_PG(14:0_16:1)	-2.3	-3.1	-1.5	-2.1	-1.5	-2.0	1.1	1.9	-1.1	1.9	1.5	3.3	-2.4	-2.8	-4.3	1.0	-2.0	-2.0	2.5	1.4	1.5
PG(32:0)_PG(16:0_16:0)	1.2	1.6	1.6	1.9	-1.0	1.1	-1.3	-1.1	-1.3	1.3	-1.1	1.1	1.3	-1.4	-1.8	-1.2	-2.9	-1.9	-1.3	-2.0	-1.8
PG(32:1)_PG(16:0_16:1)	-2.5	-3.5	-1.3	-3.0	-1.7	-2.4	1.6	2.0	1.3	1.7	1.4	2.9	-2.7	-3.5	-5.7	1.5	-2.2	-2.4	2.5	1.5	1.3
PG(32:2)_PG(14:0_18:2)	1.2	1.4	1.4	1.5	1.0	1.2	-1.1	1.0	-1.0	1.1	-1.0	1.2	-1.0	-1.4	-2.2	-1.4	-2.0	-2.3	-1.4	-2.0	-2.0
PG(32:2)_PG(16:1_16:1)	-1.9	-2.4	-1.6	-2.3	-1.1	-1.6	1.8	3.3	1.4	2.5	2.2	4.8	-2.3	-3.0	-5.7	1.5	-1.4	-2.3	3.4	1.9	1.3
PG(32:3)_PG(14:0_18:3)	1.3	1.3	1.6	1.8	1.3	1.3	-1.3	-1.1	-1.3	1.3	-1.0	1.2	-1.1	-1.9	-2.0	-1.8	-3.8	-2.6	-1.6	-2.6	-2.2
PG(34:1)_PG(16:0_18:1)	-1.3	1.1	-1.1	1.2	-1.7	-1.3	-1.1	1.2	-1.1	1.3	-1.2	-1.0	-1.5	-2.5	-2.8	-1.4	-2.6	-2.0	-1.4	-2.2	-2.2
PG(34:2)_PG(16:0_18:2)	1.3	1.6	1.5	1.6	1.1	1.2	1.0	1.2	1.1	1.3	1.1	1.3	-1.0	-1.6	-2.2	-1.3	-2.3	-2.2	-1.3	-1.9	-2.1
PG(34:2)_PG(16:1_18:1)	-3.7	-3.5	-1.7	-3.2	-1.6	-2.0	1.7	2.2	1.4	1.8	1.9	3.4	-2.3	-3.0	-4.3	2.7	-1.3	-1.4	3.3	1.9	1.6
PG(34:3)_PG(16:0_18:3)	1.5	1.9	1.7	1.9	1.2	1.3	-1.0	1.2	-1.0	1.4	1.0	1.2	-1.1	-1.8	-2.0	-1.8	-3.0	-2.4	-1.8	-2.4	-2.2
PG(34:3)_PG(16:1_18:2)	-1.1	1.2	1.2	1.2	1.2	1.3	1.0	1.1	-1.1	-1.0	1.1	1.4	-1.2	-1.3	-2.0	-1.1	-1.7	-2.2	-1.3	-1.6	-1.9
PG(36:1)_PG(16:0_20:1)	1.5	2.0	1.4	1.7	-1.2	-1.0	1.0	1.3	1.0	1.3	-1.2	-1.2	-1.1	-1.7	-1.5	-1.7	-2.4	-1.5	-1.7	-2.2	-1.8

PG(36:1)_PG(18:0_18:1)	-2.9	-2.2	-1.9	-1.5	-2.8	-2.4	-1.7	1.0	-1.5	1.1	-1.8	-1.3	-2.1	-2.2	-2.5	-1.2	-1.7	-1.6	1.1	-1.4	-1.3
PG(36:2)_PG(18:0_18:2)	1.3	1.4	1.1	1.3	-1.4	-1.3	1.0	1.3	1.1	1.6	-1.1	1.2	1.1	-1.4	-1.4	-1.1	-1.4	-1.1	1.0	-1.1	1.1
PG(36:2)_PG(18:1_18:1)	-2.0	-1.6	-1.6	-1.4	-2.8	-2.4	1.1	1.7	-1.1	1.1	-1.6	-1.3	-2.8	-2.9	-2.9	-1.2	-2.1	-1.7	-1.0	-1.9	-1.6
PG(36:3)_PG(18:1_18:2)	1.3	1.9	1.2	1.4	-1.3	-1.1	1.1	1.4	1.1	1.3	-1.1	1.0	-1.2	-2.0	-2.2	-1.4	-2.2	-1.7	-1.6	-2.0	-1.9
PG(36:4)_PG(18:1_18:3)	1.6	2.2	1.5	1.7	-1.1	-1.0	1.1	1.3	1.1	1.4	-1.0	1.0	-1.4	-2.1	-2.3	-2.0	-2.9	-2.1	-2.2	-2.6	-2.2
PG(36:4)_PG(18:2_18:2)	1.3	1.7	1.5	1.6	1.1	1.2	1.1	1.4	1.3	1.5	1.2	1.6	-1.1	-1.5	-2.2	-1.3	-1.7	-2.0	-1.3	-1.6	-1.7
PG(36:5)_PG(18:2_18:3)	1.6	1.9	1.9	2.0	1.2	1.3	-1.0	1.3	1.1	1.6	1.3	1.6	-1.2	-1.7	-2.3	-1.9	-2.8	-2.1	-1.8	-2.1	-2.0
PG(36:6)_PG(18:3_18:3)	1.8	2.0	2.2	2.5	1.6	1.6	-1.2	1.2	1.1	1.8	1.3	1.4	-1.5	-2.3	-2.3	-3.0	-4.7	-2.9	-2.4	-3.3	-2.6
PI(32:0)_PI(16:0_16:0)	-1.1	1.5	1.0	1.3	-1.4	-1.3	-1.2	1.2	-1.0	1.1	-1.5	1.0	-1.2	-2.1	-2.0	-1.4	-2.3	-2.2	-1.5	-2.5	-1.5
PI(32:2)_PI(14:0_18:2)	-1.2	1.5	1.2	1.3	1.1	-1.0	-1.1	-1.2	-1.2	-1.1	-1.1	1.2	1.1	-1.3	-2.0	1.1	-1.7	-2.4	-1.6	-1.7	-1.6
PI(32:3)_PI(14:0_18:3)	1.2	1.8	1.4	2.1	1.0	1.0	-1.3	-1.2	-1.3	-1.0	-1.2	1.1	-1.1	-1.5	-1.8	-1.7	-2.6	-2.1	-2.3	-3.2	-1.7
PI(34:1)_PI(16:0_18:1)	1.2	2.1	-1.3	1.2	-2.3	-1.8	-1.2	1.1	-1.2	1.2	-1.5	-1.3	-1.0	-2.3	-2.4	-1.4	-2.3	-1.6	-2.0	-2.3	-1.8
PI(34:2)_PI(16:0_18:2)	1.3	1.9	1.4	1.7	-1.0	-1.1	-1.1	1.1	-1.1	1.3	1.0	1.3	1.1	-1.6	-2.3	-1.4	-2.3	-2.2	-1.5	-2.0	-1.7
PI(34:3)_PI(16:0_18:3)	1.4	2.1	1.6	1.9	1.5	1.4	-1.2	1.0	-1.2	1.2	-1.0	1.2	1.0	-1.6	-1.6	-1.7	-3.0	-2.4	-2.0	-2.5	-1.8
PI(34:3)_PI(16:1_18:2)	1.4	1.8	1.5	1.6	1.4	1.6	-1.2	1.1	-1.1	1.2	-1.0	1.3	23.4	1.9	19.3	14.0	10.5	13.5	13.4	13.4	14.7
PI(36:3)_PI(18:1_18:2)	1.3	1.7	1.4	1.5	1.0	1.1	1.0	1.1	1.1	1.2	1.0	1.2	-1.0	-1.6	-1.9	-1.3	-2.2	-1.9	-1.6	-2.0	-1.8
PI(36:4)_PI(18:1_18:3)	1.4	2.0	1.6	1.7	1.2	1.3	-1.1	1.1	-1.0	1.3	-1.0	1.2	-1.1	-1.8	-1.8	-1.7	-2.8	-2.3	-2.0	-2.4	-2.0
PI(36:4)_PI(18:2_18:2)	1.4	1.8	1.3	1.5	1.1	1.2	1.1	1.3	1.3	1.4	1.2	1.5	1.0	-1.6	-2.2	-1.2	-1.6	-1.9	-1.3	-1.7	-1.7
PI(36:5)_PI(18:2_18:3)	1.3	1.8	1.4	1.7	1.1	1.2	-1.2	1.1	-1.0	1.0	-1.3	-1.0	-1.1	-1.2	-1.1	-1.7	-1.8	-1.7	-1.8	-2.0	-1.3
PI(36:6)_PI(18:3_18:3)	1.8	2.2	2.0	2.5	1.5	1.5	-1.2	1.2	-1.0	1.5	1.2	1.4	-1.3	-1.9	-1.9	-2.9	-3.9	-2.4	-2.5	-3.1	-2.0
PS(34:3)_PS(16:0_18:3)	1.2	2.0	1.4	1.9	1.2	1.3	-1.1	1.1	1.0	1.3	-1.1	1.3	1.0	-1.4	-1.9	-1.3	-1.9	-2.5	-1.8	-2.1	-1.9
PS(34:3)_PS(16:1_18:2)	-1.7	-1.4	-1.4	-1.5	-1.3	-1.3	1.2	2.0	1.2	1.6	-1.0	1.6	-1.7	-2.0	-2.1	1.2	-1.2	-1.7	1.6	1.2	1.0
PS(36:1)_PS(18:0_18:1)	1.1	1.9	-1.0	1.1	-1.8	-1.6	1.0	1.1	-1.2	1.1	-1.5	-1.4	-1.2	-1.9	-1.7	-1.2	-2.1	-1.4	-1.9	-1.8	-1.5
PS(36:4)_PS(18:2_18:2)	1.0	1.7	-1.0	1.4	-1.2	1.0	1.0	1.4	1.1	1.1	-1.4	1.1	1.1	-1.1	-1.5	1.1	1.0	-1.7	-1.1	-1.3	-1.4
PS(36:5)_PS(18:2_18:3)	1.3	2.3	1.2	1.7	1.0	1.3	1.0	1.6	1.2	1.4	-1.1	1.3	-1.1	-1.5	-1.8	-1.4	-1.5	-2.1	-1.6	-1.7	-1.7
DGDG(32:2)_DGDG(14:0_18:2)	-1.3	-1.2	1.2	2.3	1.0	1.1	1.2	-1.1	-1.2	-1.5	2.0	2.1	-1.7	-1.0	-3.5	-1.0	-1.5	-1.8	-1.6	-3.5	-1.9
DGDG(34:1)_DGDG(16:0_18:1)	1.1	1.5	1.3	1.5	1.1	1.2	-1.2	-1.1	-1.1	-1.0	-1.2	1.0	1.0	-1.3	-1.7	-1.3	-1.9	-2.1	-1.6	-2.0	-2.0
DGDG(34:2)_DGDG(16:0_18:2)	1.0	1.4	1.2	1.3	1.0	1.1	-1.2	-1.1	-1.1	1.0	-1.1	1.1	1.1	-1.3	-1.7	-1.1	-1.7	-1.9	-1.4	-1.7	-1.7
DGDG(34:3)_DGDG(16:0_18:3)	1.2	1.6	1.5	1.7	1.2	1.3	-1.3	-1.1	-1.1	1.1	-1.1	1.1	1.0	-1.5	-1.7	-1.5	-2.4	-2.2	-1.7	-2.2	-1.9
DGDG(34:3)_DGDG(16:1_18:2)	1.4	1.9	1.6	1.7	1.3	1.4	-1.2	-1.0	-1.0	1.4	1.0	1.3	1.0	-1.7	-1.8	-1.7	-2.8	-2.3	-1.9	-2.2	-1.9
DGDG(36:2)_DGDG(18:0_18:2)	1.2	1.9	1.2	1.3	-1.3	-1.1	1.0	1.2	-1.0	1.3	-1.3	-1.1	-1.2	-1.8	-1.8	-1.4	-2.1	-1.8	-1.9	-1.9	-1.8

DGDG(36:2)_DGDG(18:1_18:1)	1.1	1.8	1.1	1.3	-1.4	-1.1	-1.1	1.0	-1.2	1.1	-1.4	-1.3	-1.1	-1.7	-1.6	-1.4	-2.2	-1.7	-2.0	-2.1	-1.9
DGDG(36:3)_DGDG(18:1_18:2)	1.2	1.7	1.3	1.5	1.1	1.2	-1.0	1.1	-1.0	1.2	-1.2	1.2	-1.0	-1.4	-1.7	-1.3	-1.9	-2.1	-1.6	-1.8	-1.7
DGDG(36:4)_DGDG(18:1_18:3)	-1.1	1.3	-1.1	1.1	-1.6	-1.3	-1.2	1.1	-1.2	1.1	-1.3	-1.1	-1.2	-1.9	-2.1	-1.3	-2.1	-1.8	-1.4	-2.0	-1.8
DGDG(36:4)_DGDG(18:2_18:2)	-1.3	-1.0	-1.0	1.0	-1.3	-1.1	-1.1	-1.2	-1.1	-1.5	-1.1	1.1	1.4	1.4	-1.0	1.6	1.3	1.2	1.2	-1.1	1.2
DGDG(36:5)_DGDG(18:2_18:3)	1.2	1.6	1.3	1.4	1.0	1.0	-1.0	1.0	1.1	1.2	1.0	1.3	1.3	-1.1	-1.3	1.1	-1.4	-1.4	-1.2	-1.3	-1.1
DGDG(36:6)_DGDG(18:3_18:3)	1.3	1.9	1.8	1.9	1.2	1.2	-1.1	-1.1	-1.1	1.2	-1.0	1.2	1.2	-1.3	-1.9	-1.3	-2.5	-2.3	-1.8	-2.1	-1.9
DGMG(16:0)	1.4	1.4	2.0	1.5	-1.0	1.5	-1.2	-1.2	-1.5	-1.6	-1.2	-1.2	4.3	4.4	2.6	2.6	1.5	2.2	2.5	1.9	1.5
DGMG(18:1)	1.1	1.5	-1.1	1.3	-2.0	-1.6	1.2	1.3	1.0	1.4	-1.4	-1.3	3.2	3.3	2.5	3.5	3.8	3.5	2.6	3.6	3.0
DGMG(18:2)	1.4	1.6	1.1	1.5	-3.6	-2.6	-1.2	-1.1	-1.1	1.2	-1.2	1.1	9.0	10.9	2.7	5.8	8.7	7.8	5.1	8.7	7.3
DGMG(18:3)	-1.2	1.2	-1.3	-1.1	-1.2	-1.2	-1.1	-1.0	-1.2	1.2	-1.2	1.1	6.0	5.5	4.8	6.6	6.3	4.5	4.8	7.3	6.4
MGDG(32:2)_MGDG(16:1_16:1)	-2.6	-2.8	-1.4	-3.1	-1.7	-2.7	1.5	1.6	1.2	1.6	1.4	3.1	-1.9	-2.3	-3.5	2.0	-1.4	-1.5	2.3	2.1	2.4
MGDG(34:2)_MGDG(16:1_18:1)	1.1	1.8	1.1	1.3	-1.3	-1.1	-1.1	1.1	-1.1	-1.0	-1.5	-1.2	-1.2	-1.7	-1.6	-1.4	-2.0	-1.9	-2.0	-2.2	-1.9
MGDG(36:3)_MGDG(18:0_18:3)	1.2	1.6	1.2	1.4	-1.1	-1.0	1.0	1.2	1.1	1.2	-1.1	-1.1	-1.0	-1.3	-1.2	-1.2	-1.4	-1.2	-1.4	-1.4	-1.3
MGMG(16:0)	1.4	1.9	1.4	1.5	1.1	1.4	-1.1	1.1	-1.1	1.0	-1.2	1.2	1.1	-1.1	-1.2	-1.4	-1.6	-1.6	-1.5	-1.6	-1.4
MGMG(18:2)	1.2	1.5	1.2	1.6	-1.2	1.1	1.1	1.3	1.1	1.4	-1.1	1.2	3.2	4.5	2.8	2.7	4.0	3.1	2.9	3.8	3.0
MGMG(18:3)	1.5	1.3	1.2	1.5	-1.4	-1.2	-1.1	1.1	1.0	1.2	-1.4	1.1	3.3	4.2	3.7	2.0	3.6	3.7	2.7	3.3	4.9
SQDG(32:3)_SQDG(14:0_18:3)	1.3	1.7	1.5	1.6	1.1	1.3	-1.1	1.1	-1.1	1.2	-1.2	1.1	-1.1	-1.5	-1.6	-1.6	-2.6	-2.2	-1.7	-2.0	-1.9
SQDG(34:2)_SQDG(16:0_18:2)	1.1	1.6	1.4	1.5	1.2	1.3	-1.0	1.1	-1.1	1.1	-1.1	1.1	-1.1	-1.4	-1.6	-1.3	-2.0	-2.1	-1.7	-1.8	-1.8
SQDG(34:3)_SQDG(16:0_18:3)	1.4	1.9	1.6	1.8	1.4	1.5	-1.2	1.1	-1.1	1.2	-1.1	1.0	-1.1	-1.6	-1.4	-1.9	-2.9	-2.1	-2.0	-2.3	-2.0
SQDG(36:2)_SQDG(18:0_18:2)	1.1	2.0	1.3	1.8	-1.4	-1.1	1.1	1.1	1.1	1.5	1.0	1.3	1.0	-1.4	-1.6	1.1	-1.7	-1.1	-1.7	-1.8	-1.1
SQDG(36:2)_SQDG(18:1_18:1)	-1.0	1.5	-1.1	-1.0	-2.3	-1.8	1.1	1.3	-1.2	1.0	-1.6	-1.4	-1.6	-2.1	-2.1	-1.5	-2.2	-1.5	-1.9	-2.0	-1.6
SQDG(36:3)_SQDG(18:0_18:3)	1.5	2.2	1.9	2.2	1.0	1.3	-1.0	1.1	-1.0	1.6	-1.0	-1.0	-1.1	-1.6	-1.3	-1.7	-3.0	-1.4	-2.2	-2.1	-1.7
SQDG(36:3)_SQDG(18:1_18:2)	1.2	2.0	1.1	1.3	-1.3	-1.1	1.1	1.2	-1.1	1.2	-1.4	-1.3	-1.3	-1.8	-1.8	-1.4	-2.1	-1.8	-2.1	-2.0	-2.2
SQDG(36:4)_SQDG(18:1_18:3)	1.4	2.1	1.5	1.6	-1.1	1.0	-1.0	1.2	-1.1	1.2	-1.3	-1.3	-1.4	-2.3	-1.7	-2.0	-4.0	-2.1	-2.5	-3.1	-2.3
SQDG(36:4)_SQDG(18:2_18:2)	1.2	1.7	1.3	1.4	1.1	1.2	-1.0	1.2	-1.1	1.3	-1.0	1.2	-1.1	-1.6	-1.9	-1.4	-2.2	-2.1	-1.6	-1.7	-1.8
SQDG(36:5)_SQDG(18:2_18:3)	1.4	1.8	1.7	1.8	1.3	1.4	-1.2	1.0	-1.1	1.4	1.1	1.3	-1.1	-1.8	-1.8	-1.8	-3.3	-2.1	-2.0	-2.3	-2.1
SQDG(36:6)_SQDG(18:3_18:3)	1.7	2.8	2.5	2.8	1.6	1.7	-1.3	1.1	-1.0	2.2	1.4	1.6	-1.2	-2.3	-2.6	-2.7	-5.9	-3.0	-3.3	-3.0	-2.8
SQMG(16:0)	1.0	1.3	1.2	1.6	-1.2	1.1	-1.1	1.1	1.0	1.4	-1.1	1.0	3.2	4.3	2.9	3.0	3.8	3.0	2.8	3.8	2.8
SQMG(18:0)	-1.1	2.6	1.6	1.7	-2.5	-1.4	-1.1	1.6	2.0	3.7	-1.6	-1.6	1.8	5.6	12.4	1.8	6.9	19.7	1.1	12.1	11.5
SQMG(18:2)	1.1	1.4	1.2	1.6	-1.2	1.1	1.1	1.3	1.1	1.6	1.0	1.2	3.0	3.8	2.3	2.8	3.7	2.9	2.8	3.8	2.6
SQMG(18:3)	1.2	1.4	1.5	2.1	-1.1	1.2	-1.1	1.1	-1.0	1.6	1.0	1.3	3.2	4.0	2.1	2.4	2.6	2.3	2.5	3.1	2.3

DAG(14:0_18:2)	1.2	1.7	1.1	1.2	1.2	1.3	-1.2	1.1	-1.5	-1.3	-1.5	1.0	1.0	1.1	-1.1	-1.3	-1.5	-2.0	-1.5	-1.4	-1.4
DAG(14:0_18:3)	1.2	2.0	2.2	2.0	1.8	1.8	-1.6	-1.1	-1.4	1.0	-1.4	1.1	-1.0	1.0	1.2	-2.0	-3.1	-2.1	-2.2	-2.0	-1.4
DAG(16:0_16:0)	1.0	1.7	1.0	1.0	-1.4	-1.3	-1.4	-1.1	-1.8	-1.3	-2.4	-1.4	1.3	1.6	1.9	-1.1	-1.2	1.1	-1.4	1.2	1.7
DAG(16:0_18:0)	1.2	1.5	1.2	-1.2	-1.2	-1.2	-1.5	-1.0	-1.6	-1.2	-2.0	1.1	1.3	1.4	1.4	-1.4	-1.3	-1.3	-1.1	1.4	1.7
DAG(16:0_18:1)	1.8	2.6	1.1	1.1	-1.7	-1.6	-1.0	1.5	-1.3	1.0	-1.9	-1.7	-1.1	-1.6	-1.4	-1.9	-2.2	-1.5	-1.8	-1.7	-1.5
DAG(16:0_18:2)	1.6	2.2	1.5	1.4	1.0	1.1	-1.1	1.3	-1.1	1.1	-1.3	1.0	1.2	-1.0	-1.2	-1.5	-1.6	-1.7	-1.4	-1.3	-1.3
DAG(16:0_18:3)	1.7	2.8	1.9	1.8	1.4	1.4	-1.3	1.2	-1.3	1.2	-1.4	1.0	-1.0	-1.2	-1.1	-2.3	-2.9	-2.1	-2.4	-1.8	-1.5
DAG(16:1_18:1)	-1.6	-1.3	-1.0	1.1	-2.3	-2.4	-1.1	1.3	-1.3	1.0	-1.5	1.1	-1.3	1.3	-1.8	1.1	1.0	-1.2	1.2	1.3	1.5
DAG(16:1_18:2)	1.1	1.3	1.4	1.4	1.1	1.2	-1.2	1.1	-1.6	-1.2	-1.2	1.1	-1.3	1.0	-1.3	-1.7	-2.2	-1.8	-1.5	-1.6	-1.5
DAG(18:0_18:0)	1.1	1.5	1.2	-1.1	1.1	-1.2	-1.9	-1.0	-1.7	-1.3	-1.8	-1.1	1.2	1.1	1.1	-1.7	-1.9	-1.8	-1.3	-1.1	1.2
DAG(18:0_18:1)	1.4	2.1	-1.2	1.0	-2.7	-2.3	-1.2	1.3	-1.5	-1.0	-2.0	-1.8	-1.0	-1.5	-1.3	-1.7	-1.9	-1.0	-1.7	-1.6	-1.1
DAG(18:0_18:2)	2.2	2.8	1.6	1.7	-1.4	-1.2	1.3	1.8	1.1	1.8	-1.1	1.1	1.1	-1.2	-1.1	-1.5	-1.6	1.2	-1.4	-1.1	1.2
DAG(18:0_18:3)	2.3	3.6	1.9	2.3	1.0	1.1	1.0	1.7	-1.1	1.8	-1.1	-1.0	-1.1	-1.2	1.0	-2.4	-2.4	-1.2	-2.2	-1.6	-1.1
DAG(18:1_18:1)	1.8	2.7	-1.0	1.0	-2.2	-2.1	1.0	1.7	-1.2	1.3	-1.6	-1.6	-1.2	-2.1	-1.6	-2.1	-2.4	-1.2	-1.9	-1.7	-1.2
DAG(18:1_18:2)	1.9	2.8	1.2	1.3	-1.5	-1.4	1.2	1.8	-1.0	1.3	-1.5	-1.3	-1.1	-1.6	-1.5	-1.8	-2.0	-1.4	-1.7	-1.6	-1.4
DAG(18:1_18:3)	2.0	3.4	1.7	1.8	-1.1	-1.1	-1.0	1.6	-1.2	1.4	-1.4	-1.3	-1.1	-1.6	-1.3	-2.4	-3.1	-1.7	-2.3	-2.0	-1.5
DAG(18:1_20:1)	2.3	3.1	1.3	1.2	-1.8	-1.9	1.2	1.8	1.0	1.3	-1.4	-1.8	-1.3	-2.2	-1.7	-2.5	-2.8	-1.3	-2.2	-2.2	-1.7
DAG(18:2_18:2)	1.6	2.3	1.5	1.5	1.1	1.2	-1.1	1.4	-1.0	1.2	-1.2	1.2	1.0	-1.2	-1.4	-1.7	-2.0	-1.8	-1.7	-1.5	-1.3
DAG(18:2_18:3)	1.7	2.6	2.2	2.0	1.6	1.7	-1.3	1.2	-1.1	1.3	-1.1	1.3	1.0	-1.2	-1.2	-2.1	-2.9	-2.0	-2.1	-1.8	-1.5
DAG(18:2_20:1)	2.5	3.3	1.7	1.8	-1.1	1.1	1.4	2.1	1.3	1.7	-1.0	1.0	-1.2	-1.6	-1.4	-2.1	-2.0	-1.3	-1.8	-1.6	-1.5
DAG(18:2_20:2)	2.2	2.4	2.1	1.7	1.3	1.3	-1.2	1.3	-1.1	1.4	1.1	1.2	1.1	-1.3	-1.3	-2.4	-2.9	-1.6	-1.6	-1.6	-1.4
DAG(18:2_22:0)	2.0	2.4	1.7	1.8	-1.0	-1.2	1.3	1.5	1.1	1.4	-1.1	1.0	1.4	1.1	-1.4	-1.1	-1.4	-1.5	-1.1	-1.2	-1.1
DAG(18:2_22:2)	1.1	1.0	-1.1	-1.5	-1.1	-1.9	-1.6	-1.3	-1.3	-1.5	-1.7	-1.4	1.3	-1.2	-1.0	-1.3	-1.3	-1.6	-1.0	-1.2	1.3
DAG(18:2_24:0)	5.2	6.4	4.4	4.9	2.9	3.1	2.0	2.6	2.1	3.3	2.6	3.5	1.6	-1.1	-1.5	-1.6	-2.2	-1.7	-1.5	-1.6	-1.4
DAG(18:2_24:1)	5.8	7.2	5.5	5.6	3.4	4.1	2.1	3.2	2.3	3.8	2.6	3.1	1.3	-1.2	-1.3	-2.1	-2.9	-1.7	-1.8	-1.9	-1.7
DAG(18:3_18:3)	1.6	2.7	2.6	2.5	1.9	1.9	-1.7	-1.0	-1.4	1.2	-1.3	1.2	1.1	-1.0	1.2	-2.6	-3.8	-2.2	-2.5	-2.0	-1.4
DAG(18:3_20:1)	2.9	4.2	2.4	2.5	1.3	1.5	1.1	2.0	1.2	2.0	1.1	1.1	-1.3	-1.9	-1.4	-3.4	-3.7	-1.6	-2.7	-2.3	-2.0
DAG(18:3_22:0)	1.6	1.8	1.4	1.4	-1.4	-1.7	-1.4	-1.3	-1.2	-1.1	-1.3	-1.3	1.8	1.1	-1.3	-1.3	-1.5	-1.2	-1.3	-1.3	1.0
DAG(18:3_22:1)	2.2	3.5	1.6	1.8	-1.0	-1.1	-1.4	1.3	-1.2	1.1	-1.5	-1.3	1.3	-1.1	1.1	-2.4	-2.1	-1.4	-2.1	-1.7	-1.1
DAG(18:3_24:0)	4.3	6.4	4.5	5.4	2.3	2.9	1.4	2.0	1.9	3.4	2.2	2.7	1.4	-1.4	-1.6	-2.2	-3.2	-1.7	-2.3	-2.2	-1.8
DAG(18:3_24:1)	4.8	7.2	4.9	5.7	2.7	3.3	1.4	2.6	1.7	2.9	1.7	2.0	1.1	-1.3	-1.1	-3.1	-3.9	-1.8	-2.5	-2.6	-1.9

ASG 16:0-Glc-campesterol	-1.8	-1.3	-1.4	-1.3	-1.2	-1.4	-2.5	-1.7	-2.3	-1.9	-1.9	-1.3	2.0	1.3	-1.3	1.5	-1.3	-2.0	1.5	-1.1	-1.2
ASG 16:0-Glc-sitosterol	-1.7	-1.2	-1.4	-1.4	-1.3	-1.4	-2.4	-2.0	-2.2	-2.0	-1.8	-1.3	2.7	1.5	-1.2	1.9	1.0	-1.6	1.7	1.1	-1.0
ASG 18:1-Glc-campesterol	-1.1	1.5	-1.1	1.1	-1.2	-1.2	-1.8	-1.1	-1.6	-1.2	-1.3	-1.2	1.3	-1.4	-1.8	-1.2	-2.0	-2.1	-1.3	-1.7	-1.8
ASG 18:1-Glc-sitosterol	1.2	2.0	-1.1	1.1	-1.3	-1.3	-1.9	-1.3	-1.7	-1.2	-1.5	-1.1	2.4	-1.0	-1.5	1.0	-1.6	-1.8	-1.1	-1.4	-1.3
ASG 18:2-Glc-campesterol	-1.6	-1.1	-1.6	-1.5	-1.2	-1.5	-2.0	-1.6	-1.8	-1.8	-1.7	-1.1	1.8	1.3	-1.7	1.4	1.1	-2.4	1.2	1.1	-1.3
ASG 18:2-Glc-sitosterol	-1.7	-1.4	-1.7	-1.6	-1.3	-1.5	-2.3	-1.9	-1.7	-1.8	-1.6	-1.2	2.5	1.5	-1.2	1.9	1.5	-1.5	1.8	1.3	1.0
ASG 18:3-Glc-campesterol	-1.7	-1.1	-1.5	-1.2	-1.2	-1.4	-2.3	-1.7	-2.2	-2.0	-2.0	-1.3	1.5	1.1	-1.5	1.2	-1.3	-2.3	1.0	-1.4	-1.4
ASG 18:3-Glc-sitosterol	-1.6	-1.2	-1.4	-1.2	-1.3	-1.4	-2.3	-2.0	-2.0	-1.8	-1.9	-1.3	2.2	1.5	-1.2	1.6	1.1	-1.7	1.4	-1.0	-1.1
ASG 20:1-Glc-sitosterol	-1.0	1.7	-1.1	1.0	-1.5	-1.4	-2.2	-1.7	-1.8	-1.4	-1.5	-1.2	2.6	1.1	-1.4	1.2	-1.5	-1.4	-1.1	-1.3	-1.2
ASG 22:1-Glc-sitosterol	-1.7	-1.3	-1.1	-1.2	-1.2	-1.6	-2.5	-1.9	-2.4	-1.5	-1.9	-1.3	2.3	1.2	-1.2	1.6	-1.8	-1.9	1.6	-1.1	1.0
Cer(t18:0_22:0)	-1.7	1.3	-2.6	-1.1	-3.0	-1.2	-2.4	-1.3	-3.1	-1.6	-3.4	-1.6	1.7	1.2	1.1	1.2	1.0	-1.1	-1.0	-1.2	-1.3
Cer(t18:0_22:1)	-1.0	3.2	-1.3	2.2	-1.6	1.7	-2.0	1.9	-1.8	2.0	-2.0	1.3	-1.1	-1.3	-1.2	-2.3	0.5	-1.5	-1.9	-1.5	-1.5
Cer(t18:0_22:1-OH)	-1.6	1.6	-2.3	1.1	-1.9	-1.0	-2.1	1.0	-2.2	-1.5	-3.3	-1.7	1.5	1.4	1.5	1.1	1.4	-1.2	-1.0	-1.1	-1.1
Cer(t18:0_23:0-OH)	-2.7	-1.5	-2.2	-1.1	-1.8	-1.7	-2.7	-1.4	-2.5	-1.8	-1.8	-1.5	1.4	1.3	-1.1	1.5	1.1	-1.1	1.6	-1.2	1.1
Cer(t18:0_24:0)	-1.9	-1.4	-3.0	-2.6	-3.7	-3.1	-2.2	-1.8	-2.7	-3.1	-4.0	-3.4	1.4	-1.0	-1.2	1.2	1.0	-1.3	1.1	-1.3	-1.3
Cer(t18:0_24:1)	1.1	1.5	-1.2	-1.1	-1.7	-1.7	-1.8	-1.1	-1.7	-1.5	-2.0	-2.0	1.0	-1.4	-1.4	-1.8	-2.1	-1.7	-1.6	-1.9	-1.7
Cer(t18:0_24:1-OH)	-1.8	-1.3	-2.2	-2.2	-2.7	-2.7	-2.0	-1.4	-2.2	-2.8	-2.7	-3.1	1.6	1.4	1.3	1.4	1.4	1.3	1.5	1.1	1.1
Cer(t18:0_25:0-OH)	-1.2	-1.0	-1.2	-1.6	-1.7	-2.0	-1.7	-1.5	-1.8	-2.1	-1.6	-1.8	1.3	-1.2	-1.6	-1.1	-1.9	-1.5	-1.1	-1.6	-1.5
Cer(t18:0_25:1-OH)	-2.3	-2.5	-1.9	-2.0	-2.5	-3.1	-3.0	-2.1	-2.5	-2.6	-1.9	-1.9	1.7	2.1	-1.1	1.3	1.6	1.2	2.0	1.6	1.5
Cer(t18:0_26:0)	-1.7	-1.2	-2.2	-2.2	-3.1	-3.3	-2.0	-1.5	-2.5	-2.7	-3.3	-2.8	1.4	-1.1	-1.4	1.2	-1.3	-1.5	1.1	-1.3	-1.2
Cer(t18:0_26:1)	-1.1	-1.0	-1.3	-1.6	-1.8	-2.4	-1.6	-1.4	-1.9	-2.2	-2.1	-2.5	1.0	-1.6	-1.7	-1.5	-2.4	-1.9	-1.3	-2.1	-1.7
Cer(t18:0_26:1-OH)	-2.0	-1.6	-2.1	-2.2	-2.6	-2.8	-2.7	-2.1	-2.3	-2.9	-2.4	-2.1	1.9	1.7	1.1	1.4	1.6	1.1	1.5	1.3	1.4
Cer(t18:1_22:0)	1.9	5.0	1.3	2.6	-1.3	1.7	-1.3	1.8	-1.3	1.8	-1.4	1.5	1.1	-1.4	-1.3	-2.3	-2.2	-1.5	-2.6	-2.0	-1.5
Cer(t18:1_22:0-OH)	1.0	2.2	-1.1	1.4	-1.2	-1.1	-1.7	1.2	-1.8	1.1	-1.5	-1.2	1.5	1.2	1.2	-1.2	-1.4	-1.1	-1.2	-1.1	1.1
Cer(t18:1_23:0-OH)	1.0	1.9	1.3	2.0	-1.1	-1.0	-1.2	1.9	-1.7	-1.0	1.0	1.4	1.0	1.1	-1.7	-1.2	-2.0	-1.5	1.1	-1.8	-1.1
Cer(t18:1_24:0)	1.2	2.0	-1.1	1.3	-1.7	-1.3	-1.8	1.0	-2.1	-1.0	-2.1	-1.2	-1.1	-1.7	-1.9	-2.4	-3.2	-2.3	-2.1	-2.2	-1.7
Cer(t18:1_24:0-OH)	1.0	1.4	-1.1	1.1	-1.3	-1.3	-1.9	1.0	-1.9	-1.0	-1.8	-1.2	1.6	1.1	1.0	-1.2	-1.7	-1.3	1.1	-1.0	1.1
Cer(t18:1_24:1-OH)	1.7	2.6	1.7	1.5	1.1	1.0	-1.3	1.4	-1.2	1.4	-1.3	-1.0	1.1	-1.3	-1.1	-2.0	-2.6	-1.7	-1.7	-1.4	-1.2
Cer(t18:1_25:0-OH)	-1.2	-1.2	-1.1	1.1	-1.2	-1.4	-2.5	-1.6	-1.4	1.0	-1.7	1.0	1.2	-1.2	-1.5	-1.7	-1.7	-2.1	-1.1	-1.3	-1.0
Cer(t18:1_26:0)	1.8	3.1	1.9	2.1	1.0	1.0	-1.4	1.4	-1.1	2.1	-1.2	1.3	-1.1	-1.9	-2.0	-2.9	-4.2	-2.5	-2.5	-1.9	-1.6
Cer(t18:1_26:0-OH)	-1.0	1.2	1.3	1.2	-1.2	-1.4	-1.7	-1.2	-1.4	1.2	-1.5	-1.0	1.4	-1.0	-1.2	-1.2	-1.7	-1.6	-1.0	1.0	1.1

HexCer(d18:1_16:0-OH)	-1.2	1.4	-1.2	-1.3	-1.5	-1.5	-1.7	-1.2	-1.6	-1.9	-2.2	-1.8	1.5	1.4	1.3	1.0	1.1	-1.1	-1.2	-1.1	1.1
HexCer(d18:1_20:0-OH)	-1.1	1.3	-1.2	-1.2	-1.3	-1.4	-1.4	-1.1	-1.4	-1.5	-1.3	-1.4	1.5	1.3	-1.0	1.3	1.1	-1.1	1.2	1.0	-1.1
HexCer(d18:1_24:0-OH)	-1.0	1.3	-1.1	-1.2	-1.4	-1.9	-1.5	-1.0	-1.4	-1.4	-1.5	-2.0	1.8	1.3	1.1	1.2	-1.0	1.0	1.4	1.0	1.1
HexCer(d18:2_14:0)	1.7	2.4	1.5	1.6	-1.1	1.1	-1.5	1.2	-1.1	1.2	-1.2	-1.2	1.3	-1.3	-1.4	-1.8	-2.1	-1.5	-1.5	-1.7	-1.8
HexCer(d18:2_14:0-OH)	1.2	1.6	1.1	1.1	-1.3	-1.1	-1.5	1.1	-1.7	-1.1	-1.5	-1.3	1.1	-1.2	-1.2	-1.8	-2.3	-1.4	-1.4	-1.5	-1.4
HexCer(d18:2_16:0)	2.0	3.6	2.3	2.8	1.2	1.5	-1.3	1.5	1.4	1.6	-1.3	1.4	1.4	1.3	1.1	-1.9	-1.3	-1.4	-1.7	-1.4	1.0
HexCer(d18:2_16:0-OH)	1.0	1.7	-1.1	-1.1	-1.4	-1.3	-1.6	-1.0	-1.6	-1.5	-2.3	-1.6	1.3	1.2	1.3	-1.2	-1.3	-1.3	-1.3	-1.1	1.1
HexCer(d18:2_16:1-OH)	-1.2	1.3	-1.2	-1.2	-1.4	-1.5	-1.6	1.0	-1.9	-1.2	-1.6	-1.6	1.1	1.0	1.1	-1.3	-1.6	-1.0	-1.2	1.0	-1.0
HexCer(d18:2_18:0-OH)	-1.1	1.6	1.0	-1.0	-1.1	-1.1	-1.7	-1.0	-1.6	-1.3	-2.0	-1.2	1.3	1.2	1.2	-1.1	-1.3	-1.4	-1.3	-1.0	1.2
HexCer(d18:2_18:1-OH)	-1.3	1.3	-1.1	-1.1	-1.5	-1.6	-1.8	-1.1	-1.6	-1.6	-2.1	-1.5	1.4	1.6	1.3	1.0	1.1	-1.1	-1.0	1.1	1.4
HexCer(d18:2_20:0-OH)	1.1	1.6	1.1	1.0	-1.1	-1.1	-1.5	1.1	-1.5	-1.0	-1.5	-1.2	1.3	-1.0	-1.1	-1.3	-1.7	-1.4	-1.2	-1.1	-1.2
HexCer(d18:2_20:1-OH)	-1.0	1.8	1.1	1.1	-1.1	-1.1	-1.7	-1.0	-1.6	-1.3	-2.0	-1.3	1.1	-1.0	1.1	-1.5	-1.8	-1.7	-1.7	-1.4	-1.1
HexCer(d18:2_22:0-OH)	1.3	1.8	1.1	1.1	-1.2	-1.3	-1.4	1.2	-1.4	-1.0	-1.5	-1.3	1.4	1.0	1.0	-1.2	-1.5	-1.2	-1.0	-1.1	1.0
HexCer(d18:2_22:1-OH)	1.3	1.9	1.3	1.3	1.1	1.0	-1.4	1.1	-1.4	1.0	-1.4	-1.3	1.3	-1.1	1.0	-1.5	-2.0	-1.4	-1.4	-1.3	-1.3
HexCer(d18:2_23:0-OH)	1.1	1.5	1.4	1.3	-1.1	-1.2	-1.4	1.2	-1.2	1.1	-1.4	-1.1	-1.0	-1.2	-1.3	-1.6	-2.1	-1.8	-1.2	-1.4	-1.2
HexCer(d18:2_23:1-OH)	1.2	1.9	1.2	1.2	1.1	1.0	-1.6	-1.0	-1.6	-1.4	-1.3	-1.1	1.6	1.4	-1.1	-1.3	-1.3	-1.6	-1.3	-1.2	-1.2
HexCer(d18:2_24:0)	1.6	2.1	1.3	1.5	-1.0	-1.0	-1.3	1.5	-1.1	1.5	1.0	-1.1	1.2	-1.4	-1.6	-1.7	-2.0	-1.5	-1.2	-1.4	-1.7
HexCer(d18:2_24:0-OH)	1.3	1.8	1.1	1.1	-1.2	-1.2	-1.4	1.2	-1.4	-1.0	-1.5	-1.3	1.4	-1.0	1.0	-1.3	-1.7	-1.2	-1.1	-1.1	-1.1
HexCer(d18:2_24:1-OH)	1.4	2.0	1.3	1.2	-1.1	-1.1	-1.4	1.1	-1.4	-1.0	-1.5	-1.4	1.2	-1.2	-1.1	-1.7	-2.2	-1.5	-1.5	-1.5	-1.4
HexCer(d18:2_25:0-OH)	1.1	1.3	1.1	1.0	-1.1	-1.3	-1.4	1.3	-1.5	1.1	-1.5	-1.2	-1.2	-1.6	-1.5	-1.9	-2.8	-2.0	-1.3	-1.4	-1.3
HexCer(d18:2_25:1-OH)	1.2	1.5	1.2	1.2	-1.1	-1.2	-1.6	1.1	-1.9	-1.1	-1.7	-1.3	-1.0	-1.2	-1.1	-2.0	-2.8	-1.7	-1.3	-1.6	-1.2
HexCer(d18:2_26:0)	2.1	2.7	1.6	1.8	1.1	1.1	-1.1	1.6	1.0	1.6	1.1	1.2	1.2	-1.4	-1.7	-1.9	-2.2	-1.7	-1.4	-1.6	-1.6
HexCer(d18:2_26:0-OH)	1.1	1.6	1.1	1.1	-1.1	-1.2	-1.4	1.2	-1.5	-1.0	-1.5	-1.2	1.1	-1.2	-1.3	-1.4	-1.9	-1.7	-1.2	-1.3	-1.3
HexCer(d18:2_26:1-OH)	1.3	1.8	1.2	1.2	-1.1	-1.1	-1.6	1.1	-1.7	-1.1	-1.6	-1.4	1.1	-1.3	-1.1	-1.8	-2.6	-1.6	-1.5	-1.6	-1.4
HexCer(t18:0_22:0-OH)	1.2	1.6	-1.1	-1.1	1.1	-1.0	-1.4	1.2	-1.4	-1.3	-1.5	-1.3	1.5	1.2	1.1	-1.1	-1.1	-1.5	1.1	-1.0	-1.2
HexCer(t18:0_22:1-OH)	-1.3	-1.1	-2.0	-1.8	-1.3	-1.1	-1.7	-1.2	-1.8	-2.4	-1.5	-3.1	1.8	1.6	2.0	1.3	1.8	1.7	1.6	1.2	-1.5
HexCer(t18:0_24:1-OH)	-1.3	-1.0	-1.9	-1.9	-1.5	-1.6	-1.6	-1.2	-1.6	-2.3	-1.7	-2.3	1.7	1.3	1.1	1.3	1.5	-1.0	1.4	1.1	-1.3
HexCer(t18:1_16:0-OH)	1.1	1.6	-1.1	-1.1	-1.3	-1.2	-1.5	1.0	-1.5	-1.5	-1.6	-1.6	1.6	1.3	1.0	-1.0	-1.1	-1.1	1.1	-1.0	-1.3
HexCer(t18:1_18:0-OH)	1.0	1.5	-1.3	-1.2	-1.2	-1.1	-1.7	-1.2	-1.4	-1.5	-2.0	-1.4	1.6	1.2	1.2	-1.1	1.1	-1.5	-1.0	1.0	-1.1
HexCer(t18:1_20:0-OH)	1.1	1.6	1.1	1.0	-1.0	1.0	-1.7	-1.2	-1.4	-1.4	-1.7	-1.3	1.8	1.6	1.4	1.0	1.1	-1.2	-1.0	1.1	1.0
HexCer(t18:1_22:0-OH)	1.2	1.7	1.0	-1.0	-1.1	-1.1	-1.5	1.0	-1.5	-1.4	-1.6	-1.4	1.8	1.5	1.2	-1.0	1.0	-1.2	1.0	1.1	-1.1

HexCer(t18:1_22:1-OH)	1.2	1.8	1.1	1.1	-1.1	-1.1	-1.5	1.0	-1.4	-1.3	-1.6	-1.6	1.5	1.3	1.2	-1.2	-1.2	-1.2	-1.2	-1.1	-1.2
HexCer(t18:1_23:0-OH)	1.2	1.4	-1.1	-1.1	-1.2	-1.3	-1.1	1.3	-1.5	-1.2	-1.5	1.0	-1.0	-1.2	-1.6	-1.3	-1.7	-1.9	-1.2	-1.3	-1.2
HexCer(t18:1_23:1-OH)	1.1	1.5	1.1	1.1	1.1	-1.1	-1.4	1.0	-1.4	-1.1	-1.4	-1.1	1.1	1.0	-1.2	-1.4	-1.5	-1.7	-1.4	-1.1	-1.3
HexCer(t18:1_24:0)	1.3	1.5	-1.0	1.0	-1.4	-1.3	-1.6	1.1	-1.5	-1.3	-1.5	-1.7	1.1	-1.1	-1.3	-1.8	-1.5	-1.5	-1.2	-1.4	-1.7
HexCer(t18:1_24:0-OH)	1.2	1.7	-1.0	-1.0	-1.2	-1.2	-1.5	1.1	-1.5	-1.2	-1.6	-1.4	1.5	1.1	1.0	-1.2	-1.3	-1.4	-1.0	-1.1	-1.2
HexCer(t18:1_24:1-OH)	1.3	1.9	1.2	1.1	-1.1	-1.1	-1.4	1.1	-1.4	-1.2	-1.6	-1.3	1.2	1.0	-1.0	-1.5	-1.7	-1.5	-1.4	-1.3	-1.3
HexCer(t18:1_25:0-OH)	1.1	1.3	1.0	-1.1	-1.1	-1.3	-1.4	1.2	-1.4	-1.1	-1.6	-1.2	-1.2	-1.3	-1.5	-1.8	-2.0	-2.1	-1.3	-1.4	-1.4
HexCer(t18:1_25:1-OH)	1.2	1.4	1.1	-1.0	-1.1	-1.2	-1.4	1.1	-1.8	-1.2	-1.7	-1.2	-1.0	-1.2	-1.3	-1.7	-2.4	-2.0	-1.4	-1.4	-1.3
HexCer(t18:1_26:0-OH)	1.2	1.5	-1.0	1.0	-1.1	-1.2	-1.5	1.1	-1.5	-1.1	-1.7	-1.3	1.4	1.0	-1.2	-1.3	-1.5	-1.7	-1.0	-1.1	-1.2
HexCer(t18:1_26:1-OH)	1.3	1.7	1.2	1.1	-1.1	-1.1	-1.5	1.0	-1.5	-1.1	-1.8	-1.4	1.2	-1.1	-1.0	-1.7	-2.0	-1.7	-1.4	-1.4	-1.4
SG. campesteryl glucoside	-1.3	1.0	-1.3	-1.4	-1.5	-1.4	-2.0	-1.5	-1.8	-1.5	-1.6	-1.5	1.4	1.4	1.2	-1.0	-1.0	1.1	-1.1	1.2	1.1
SG. cholesteryl glucoside	1.3	1.9	-1.0	-1.0	-1.0	-1.1	-1.5	-1.1	-1.2	-1.6	-1.9	-1.2	1.2	1.0	1.5	-1.6	-1.1	-1.2	-1.8	-1.5	1.4
SG. sitosteryl glucoside	-1.7	-1.1	-1.8	-1.8	-1.6	-1.6	-2.3	-1.7	-2.1	-2.0	-2.0	-1.7	2.0	1.8	1.7	1.5	1.5	1.3	1.2	1.6	1.6
